# Climate anomalies affect annual survival rates of swifts wintering in sub‐Saharan Africa

**DOI:** 10.1002/ece3.6525

**Published:** 2020-07-06

**Authors:** Giovanni Boano, Irene Pellegrino, Mauro Ferri, Marco Cucco, Fausto Minelli, Susanne Åkesson

**Affiliations:** ^1^ Museo civico di Storia naturale Carmagnola Italy; ^2^ Department of Science and Technological Innovation, University of Piemonte Orientale. Alessandria Italy; ^3^ Associazione Ornitologi Emilia‐Romagna Bologna Italy; ^4^ Ente di gestione per i Parchi e la Biodiversità Emilia Centrale Modena Italy; ^5^ Department of Biology Center for Animal Movement Research Lund University Lund Sweden

**Keywords:** Annual survival rate, Apus apus, Apus pallidus, capture‐mark‐recapture data, climatic anomalies, drought, rainfall, ringing recoveries, wintering area

## Abstract

Several species of migratory swifts breed in the Western Palearctic, but they differ in reproductive traits and nonbreeding areas explored in Africa. We examined survival and recapture probabilities of two species of swifts by capture–mark–recapture data collected in northern Italy (Pallid Swift *Apus pallidus* in Carmagnola, Turin, and Common Swift *Apus apus* in Guiglia, Modena) in the breeding season (May–July). Apparent survival rates were relatively high (>71%), comparable to other studies of European swifts, but showed marked annual variations. We used geolocators to establish the exact wintering areas of birds breeding in our study colonies. Common Swifts explored the Sahel zone during migration and spent the winter in SE Africa, while the Pallid Swifts remained in the Sahel zone for a longer time, shifting locations southeast down to Cameroun and Nigeria later in winter. These movements followed the seasonal rains from north to south (October to December). In both species, we found large yearly differences in survival probabilities related to different climatic indices. In the Pallid Swift, wintering in Western Africa, the Sahel rainfall index best explained survival, with driest seasons associated with reduced survival. In the Common Swift, wintering in SE Africa, the El Niño–Southern Oscillation (ENSO) cycle performed significantly better than Sahel rainfall or North Atlantic Oscillation (NAO). Extreme events and precipitation anomalies in Eastern Africa during La Niña events resulted in reduced survival probabilities in Common Swifts. Our study shows that the two species of swifts have similar average annual survival, but their survival varies between years and is strongly affected by different climatic drivers associated with their respective wintering areas. This finding could suggest important ecological diversification that should be taken into account when comparing survival and area use of similar species that migrate between temperate breeding areas and tropical wintering areas.

## Graphical abstract

We examined survival and recapture probabilities of two species of swifts by capture–mark–recapture data collected in northern Italy in the breeding season, and we used geolocators to establish the exact wintering areas of birds breeding in our study colonies. Survival rates were relatively high (>71%) and showed marked annual variations related to different climatic indices. In the Pallid Swift, the Sahel rainfall index best explained survival, with driest seasons associated with reduced survival, while in the Common Swift the El Niño–Southern Oscillation (ENSO) cycle was associated with survival.

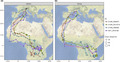

## 1 INTRODUCTION

In the Western Palearctic, several species of swifts breed in the same geographic area, where different species may form mixed colonies in natural breeding sites (Mazzotto, Cucco, & Malacarne, 1996). They may also breed in buildings constructed by humans (Cucco & Malacarne, 1987). However, swift species that mix in the breeding area differ noticeably in their biological traits and circannual timing and may spend the winter in different parts of the African continent. Traits that differ between two swifts breeding in the same area, the Common *Apus apus* and the Pallid Swift *Apus pallidus* (Pellegrino et al., 2017), include number of clutches produced per year (Cucco, Malacarne, Orecchia, & Boano, 1992), diet composition (Cucco, Bryant, & Malacarne, 1993), and moult strategy (Boano, Pellegrino, & Cucco, 2015). At the same time, they show similar movement adaptations during the nonbreeding period including continuous flight, but extending for different lengths of time, with Common Swifts staying airborne around 10 months (<1% landing; Hedenström et al., 2016), and Pallid Swifts 5 months (<1% landing; Hedenström et al., 2019). The large difference in breeding period between the two sympatric swift species could potentially originate from differences in preferred winter destinations including length of wintering periods. However, due to their strictly aerial habits (Hedenström et al., 2016, 2019), observations during winter are hard to collect, and until recently, little was known about both location of wintering areas explored (Åkesson, Klaassen, Holmgren, Fox, & Hedenström, 2012; Norevik et al., 2019) and the annual survival of these continuously flying insectivorous species.

There is substantial variation in annual survival between bird species depending on lifestyle and movement ranges, where seabirds, swifts, and allies show some of the longest life spans recorded in birds (Lack, 1968). Survival of different swift species shows very similar and high values, though variations due to different species, study conditions, and method of data analysis are present. The Common Swift survival estimates span between 76% and 81% (Baillie & Green, 1987; Dobson, 1990; Viallefont, Lebreton, Reboulet, & Gory, 1998), while in the closely related Pallid Swift the annual survival varies in the range 74%–76% (Boano, Cucco, Malacarne, & Orecchia, 1993). The larger Alpine Swift *Tachymarptis melba* survival has been estimated to 79% (Glutz von Blotzheim & Bauer, 1980). Survival estimates of swifts from the New World show similarly high values: Chimney Swift *Chaetura pelagica* between 62% and 81% (Collins, 1973; Dexter, 1979), White‐throated Swift *Aeronautes saxatalis* 80% (Collins, 1973), and Chestnut‐collared Swift *Streptoprocne rutila* between 83% and 85% (Collins, 1974).

Most survival estimates for swifts have been collected in a single or a limited number of locations. In particular, the survival of Common Swift, corresponding to one of the most widespread and abundant swift species in the Western Palearctic, has until now only been studied in Great Britain and France. Moreover, no study so far has investigated the influence of climate drivers in wintering or passage grounds on the survival of swifts. One of the few studies focusing on the effect of weather on survival solely considered the effect of local climate experienced on the breeding ground, leading to reduced adult survival associated with low temperatures in July (Thomson, Douglas‐Hhome, Furness, & Monaghan, 1996). Thus, it is important to investigate what effects climate drivers may have on the swifts’ survival in other periods of the year. In particular, this is central for conservation of the species, in order to understand under which period of the annual cycle the swifts face the largest mortality risk (Marra, Cohen, Loss, Rutter, & Tonra, 2015).

Our aim was to compare the climatic factors affecting survival of two species of swifts, the Common and the Pallid Swift, sampled among populations breeding in the same area (northern Italy), but wintering in different parts of the African continent. In the present study, we provide one of the few estimates of survival rates for adult Common Swift in Europe outside England and France, and the first study considering the effect of climate outside the breeding season. To record the wintering areas of our study populations, and to assess the nonbreeding movements of breeding adults, we used miniature data‐logger geolocators to track adult breeding Common Swifts and used information from a recent study reporting nonbreeding ranges for our Pallid Swift population (Norevik et al., 2019).

## 2 METHODS

### 2.1 Study sites and field protocols

#### 2.1.1 Pallid Swift

The study site is localized in two old buildings in the center of the town of Carmagnola (Turin, Italy) (Lat. 44.84°, Long.: 7.72° E, 239 m asl; Boano, 1974). During the study period, the colony has grown from about 30 to > 100 breeding pairs. Our main study activity was done in a subset of the colony with nests accessible from inside the private house. Here, we could inspect about 25 nest holes occupied annually by 7–20 pairs. These nests were inspected around every fortnight (from 1982 to 1986) or daily (from 1987 to 1990) for concurrent ecological and ethological studies (Boano & Cucco, 1989; Cucco & Malacarne, 1996a, 1996b, 1996c). From 1991 and thereafter, we reduced the effort to 2–3 visits per season (mainly in July) to ring all young and most adults. For survival estimation of this population, we used the data of ringed adults from 1984 to 1992, when the capture effort was higher (Boano et al., 1993), and those of a second period from 2002 to 2012, with a lower capture effort but in the same years of the Common Swift sample, so particularly suited for comparisons.

#### 2.1.2 Common Swift

The study was performed in the Regional Park of Sassi di Roccamalatina, Guiglia, Modena, in the medieval tower of Castellaro (44.39°N, 10.95°E, 490 m asl). At this breeding colony, nests were checked at least twice per year for over 22 years (from 1991 to 2012), the first time in late May, to capture breeding adults and to record number of the eggs for each pair, and again in late June to ring the chicks and adults feeding young. In the first 10 years, the nests were checked more irregularly with some years lacking, but from 2001 the monitoring was regular up to the final year 2012. Thus, the data used here are from 2001 to 2012, including only the period with regular checking. During this period, the colony increased from 17 to 51 breeding pairs (averaging 35 per year), thanks to some management of the vegetation nearby the tower and of the nest facilities (Minelli et al., 2014).

In both study sites, all birds captured were ringed with metal rings and controlled when captured again. Only adult birds were used for the analyses, because the very low philopatry of young swifts (Lack, 1956; personal observations) prevents the possibility to recapture them after fledging.

### 2.2 Tracking nonbreeding movements by geolocation

We utilized geolocation data collected in the same swift colonies where we assessed survival. We used archival light loggers (Model Intigeo W55B1 and W65B1) from Migrate Technology Ltd. (all without a stalk) to track the nonbreeding movements of adult breeding swifts. The adult breeding Common and Pallid Swifts were captured inside the nest boxes located in the building after sunset at night. We timed the catching and attachment of geolocators to the late stage of the breeding period, when the young were near to leaving the nest, but the parents were still actively feeding the young (second half of the breeding period; Åkesson, Bianco, & Hedenström, 2016; Åkesson et al., 2012). We used a full body harness (soft braided nylon string) to attach the geolocators to the bird by three loops around neck and each wing, respectively (total mass 0.7–1.3 g including harness, never exceeding 3% of the bird's body mass), as described by Åkesson et al. (2012). After capture and immediate logger attachment, the birds were released at the colony. We did not include individuals fitted with geolocators in the analysis of survival due to their documented lower return rate for logged birds to the breeding colony (Morganti et al., 2017). The wintering area utilized by the Pallid Swifts breeding at our study site was taken from the results reported in a recently published study based on geolocation (Norevik et al., 2019). For further information on capture method of Pallid Swifts, see Norevik et al. (2019).

In 2010–2012, we attached 28 geolocators to adult Common Swifts in the breeding colony in Italy, and we were able to recover 4 logged individuals. At recapture, we did not find any negative effect on plumage or skin caused by the attachment of the geolocators on the retrapped swifts.

We used the program Intiproc v.1.03 provided by the manufacturer Migrate Technology Ltd, to perform the initial linear correction function for the clock drift and extracting times for sunrise and sunset using 2 as the light threshold. We used the critical sun angle corresponding to a light‐level value of 2 on the arbitrary geolocator light scale (Migrate Technology Ltd.) minimizing the difference in latitude between pre‐ and postequinox, and at the same time minimizing the uncertainty in latitude close to the equinox for periods when the birds were stationary as defined by the estimations of longitude. We used 0.5 and 0.3 steps of critical sun angle extracted and evaluated across a range of sun angles (8–12 per bird) to define the one resulting in lowest difference in latitude between pre‐ and postequinox periods. We used the “Hill‐Ekstrom” procedure (Ekstrom, 2004) to evaluate which sun angle we should use for respective track as outlined in Åkesson et al. (2012), Åkesson et al. (2016). The sun angles used varied between −3.7° and −6.7° depending on model. We recorded minimal clock drift (0–2 min) over one year, and no consistency in the drift for our loggers. We excluded light data on latitude and longitude corresponding to ca 14 days before and after autumn and vernal equinoxes, respectively, and 21 days after and before autumn and vernal equinoxes, respectively, from the evaluations. The errors recorded by archival light‐level geolocators are influenced by geographic location, time of year, habitat type, and weather and correspond to values of 143 ± 62 km (mean ± 95% confidence interval) in terrestrial systems (Fudickar, Wikelski, & Partecke, 2011), and 186 ± 114 km (mean ± *SD*) in marine environments (Phillips, Silk, Croxall, Afanasyev, & Briggs, 2004) for latitude. The corresponding values for errors of longitude estimates are lower 50 ± 34 km (mean ± 95% confidence interval) for terrestrial environments (Fudickar et al., 2011) and 85 ± 47 km (mean ± *SD*) at sea (Phillips et al., 2004). Reports show that weather, topography, and vegetation have the strongest impact on accuracy in geolocator tracking data for terrestrial birds, leading to shading and variations in light intensity (Lisovski et al., 2012). Conversely, for swifts and other aerial birds who spend a large fraction of their time on the wing mainly weather influence the geolocator precision, resulting in typically very clean light measurements and no shading effects (Åkesson et al., 2016).

### 2.3 Mark–recapture analysis

Survival estimates derived from analysis of recaptures or resightings of living marked birds are widely used in association with proper stochastic open‐population models (Cormack, 1964; Seber, 1982; Williams, Nichols, & Conroy, 2002). Estimates derived from capture–recapture experiments should be considered as minimal (or “apparent”) survival rates, although the bias is frequently negligible for adult philopatric bird species. In this study, capture–recapture data were used in association with open‐population models (Cormack–Jolly–Seber, CJS hereafter, and related models), and associated model selection criteria (e.g., Lebreton, Burnham, Clobert, & Anderson, 1992; Nichols, 1994; Williams et al., 2002). These models produce survival estimates that are not influenced by variations in recapture probability, and therefore are more reliable than those based on return rates alone (Martin, Clobert, & Anderson, 1995; Nichols & Pollock, 1983). Survival probability (*Фi*) was defined as the probability that an animal, living at period *i*, is still alive and available for recapture at period *i* + 1, and recapture probability (*pi*) is the capture probability of an animal alive and in the population at the sampling time *i*.

According to previous considerations, survival probability complement (1–*Ф*) includes both mortality and permanent emigration from the study site and is here referred as “apparent” survival according to Thomson et al., (2009) that we follow for all the relevant terminology.

Analysis started with program U‐CARE (Choquet, Lebreton, Gimenez, Reboulet, & Pradel, 2009) to compute the goodness‐of‐fit test (GOF) of the most general model, where *Фi* and *pi* vary only with time, and other specific tests for transience and trap dependence. GOF procedures are used to identify a general statistical model “fitting” the data. Then, models making further restrictions were fitted with program MARK version 9.0 (Cooch & White, 2002; White & Burnham, 1999), including models where temporal variation in survival is modeled as a logit‐link function of specific weather conditions. The selection of the most appropriate model was based on the Akaike's information criterion (AIC) (Anderson & Burnham, 1999; Burnham, Anderson, & White, 1995). In this study, we adopted AICc values (AIC approximated for small samples) or QAICc (quasi‐AICc) when the data were overdispersed as evidenced by ĉ (the variance inflation factor) >1 (White & Burnham, 1999). If overdispersion is present, then model selection should be based on QAICc (QAICc = AICc/ĉ). We also applied analysis of deviance (ANODEV) to assess the statistical significance and the fraction of temporal variation explained by each covariate used in the model (Grosbois et al., 2008; Lebreton et al., 1992; Rolland, Barbraud, & Weimerskirch, 2008; Skalski, Hoffman, & Smith, 1993).

To investigate the relationships between survival rates and climate conditions potentially influencing swift survival, three main climatic variables were considered, that is, (1) Sahel rainfall, (2) the North Atlantic Oscillation (NAO), and (3) the El Niño–Southern Oscillation (ENSO). For this purpose, we fitted models with time constraints on capture and survival rates. The output of the program MARK provides the deviance of the model fitted to an additive constant that depends on the data set but not on the model. The effect of covariates in MARK is tested by building a linear model where the parameter estimates are constrained to be linear functions of one or more covariates. MARK allows temporal variation in survival (or capture probabilities) to be modeled as a logit‐link function of specific covariates. This procedure is better than an ordinary regression analysis of the CJS estimates over the variable of interest, because it avoids the pitfalls of the autocorrelation of estimates and the entire variance–covariance structure of the estimates is properly taken into account (Lebreton et al., 1992 and references therein).

1. Sahel Rainfall: Annual Rainfall indices for the Sahel zone comprised between 20°–10°N and 20°W–10°E were taken from Mitchell (2018). We used JJASO (June through October) means expressed in 0.1 mm of precipitation as deviations with respect to the 1900–2010 mean, so “‐83” corresponds to −0.83 cm precipitation anomaly. The index represents the quantity of rainfall from June to October (the rainy season) of each year and was applied to the cohorts of birds released in the same year, considering that survival of each cohort could be affected by the wet season immediately preceding the wintering period.

This index was found to potentially correlate with bird survival as, in a number of European trans‐Saharan migrants, precise relationships have emerged between annual breeding numbers or survival and annual rainfall in African wintering areas (Newton, 2008; Zwatrts, Bijlsma, Van der Kamp, & Wymenga, 2009). Moreover, to investigate the effect of extreme low rainfall values, the years were categorized to reflect whether or not a given rainy season was “very dry” following the numerical limits explicitly defined by Landsea, Gray, Mielke, and Berry (1997), coding the dryer years as “1” and all other years as “0” in the categorical model.

**FIGURE 1:**
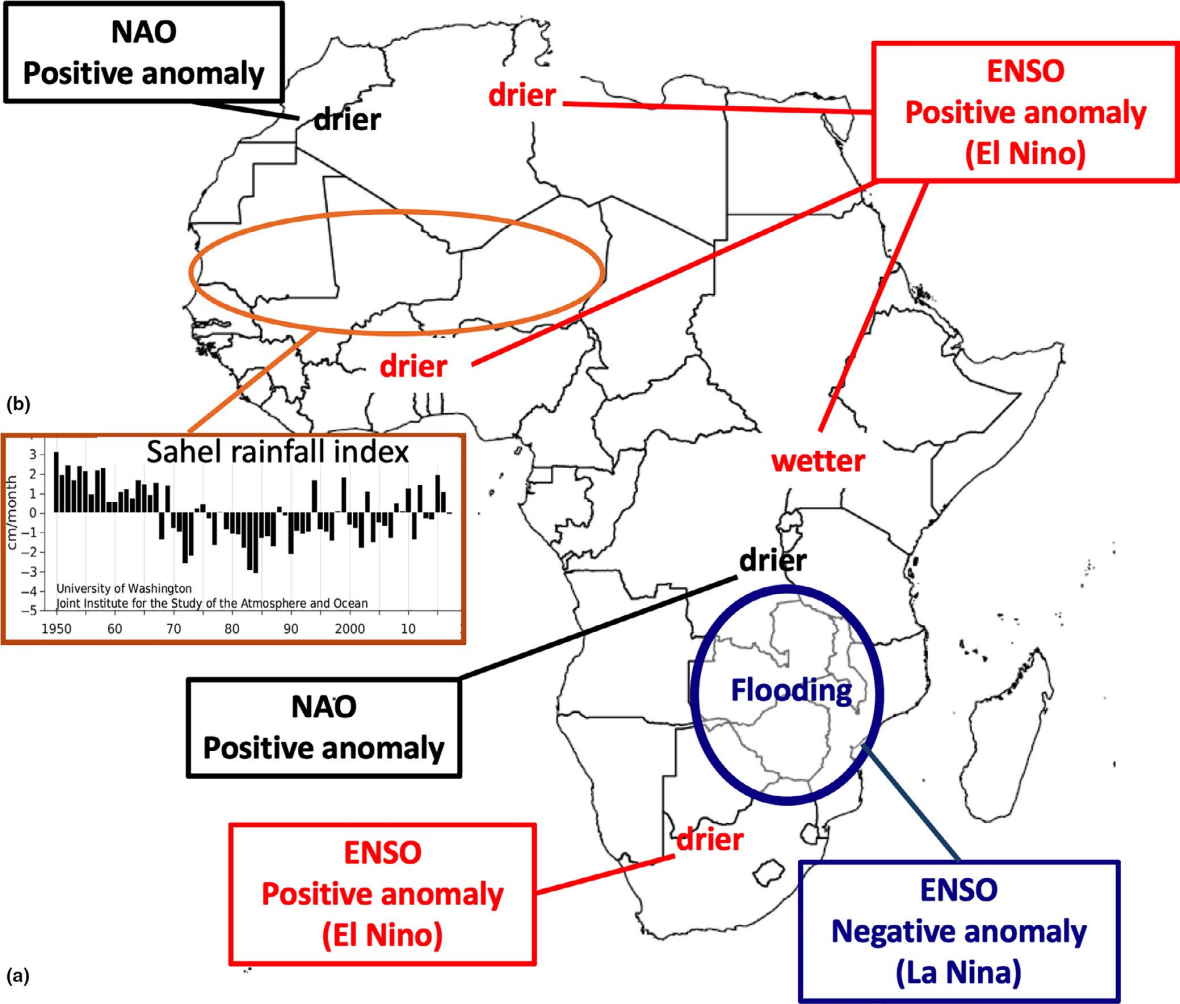
(a) Main effect of ENSO and NAO to local climate in Africa. Extensive flooding in South Africa in January and March 2008 that may be associated with the low survival of our Swifts from the breeding season 2007 to the one 2008 (modified from http://www.ncdc.noaa.gov/temp‐and‐precip/global‐maps/200801?products[]=extremes). (b) Sahel precipitation anomalies (from http://research.jisao.washington.edu/data/sahel/)

- NAO: The North Atlantic Oscillation Index is a large‐scale oscillation in atmospheric masses between the subtropical high and the polar low and is an index useful as a measure of the general climatic conditions in large parts of Europe (Hurrell, Kushnir, Ottersen, & Visbeck, 2003). Furthermore, Mediterranean precipitation is correlated with NAO, with negative anomalies for the positive phase of the oscillation and northward shifts of the storm track during the positive phases of the oscillation (Baldi, Cesarone, Carella, Crisci, & Dalu, 2004; Delitala, Cesari, Chessa, & Neil, 2000). More interestingly for our purposes, the NAO is now considered to show an association with rainfall variability and some influence on the productivity of diverse African regions, including eastern (Stige et al., 2006) and southeastern Africa (McHugh & Rogers, 2001). Values of NAO for the month from December to March are taken from https://climatedataguide.ucar.edu/sites/default/files/nao_station_djfm.txt.
- ENSO: As index of the El Nino–Southern Oscillation (ENSO) cycle, we used the Oceanic Niño Index (ONI) for the months December, January, and February. ONI is based on sea surface temperature anomalies and is defined as the three‐month (in our case Dec‐Jan‐Feb) running‐mean SST (sea surface temperature) departures in the Niño 3.4 region, based on the NOAA ERSST data. El Niño is characterized by ONI ≥ +0.5°C, while La Niña is based on ONI ≤ −0.5°C. An El Niño or La Niña episode is defined when the above thresholds are exceeded for a period of at least 5 consecutive overlapping 3‐month seasons. We used ONI data for the trimester Dec‐Jan‐Feb downloaded from http://www.cpc.noaa.gov/products/analysis_monitoring/ensostuff/ensoyears.shtml. Because of strong effect in an area visited by Common Swift in winter (see Figure 1), we also tested a categorical model obtained simply by coding the La Niña years as “1” and all other years as “0.”

The complex effects of these climatic drivers on different African regional climates are summarized in Figure 1.

## 3 RESULTS

### 3.1 Pallid Swift

#### 3.1.1 Mark–recapture analysis

In the first study period (1984–1992), a sample of 106 breeding Pallid Swifts provided 194 capture events (counting only one recapture per year) (Table 1). The GOF test of the general CJS model *Ф*(*t*)*p*(*t*) (*χ* ^2^ = 4.777; *df* = 20; *p* = .999) suggested that the CJS model's assumptions were very well meet; ĉ estimated by *χ* ^2^/*df* was < 1 so no correction was needed. Specific test for transient (U‐Care test 3.SR) and for trap dependence (U‐Care TEST2.CT) were not significant (statistic for transient = 0.005, *p* = .996; signed statistic for trap dependence = 0.154, *p* = .877).

**TABLE 1:** Pallid Swift *Apus pallidus*, period 1984–1992: results of the comparison of the general and related (nested) capture–recapture models (in bold results with delta AICc < 2)

*N*	Model	np	AICc	ΔAICc	Model Weights	Deviance	*R* ^2^
**1**	**{** *Ф* **(Sahel “very dry” vs. “other” years) *p*(*t*)}**	**10**	**323.4**	**0.000**	**0.482**	**82.344**	.557
**2**	**{** *Ф* **(Sahel rainfall index) *p*(*t*)}**	**10**	**324.6**	**1.237**	**0.260**	**83.582**	.434
3	{*Ф* (c) *p*(*t*)}	9	326.7	3.322	0.092	87.939	
4	{*Ф* (NAO) *p*(*t*)}	10	327.4	3.983	0.066	86.327	.160
5	{*Ф* (ONI) *p*(*t*)}	10	328.1	4.761	0.045	87.105	.083
6	{*Ф* (la Niña years) *p*(*t*)}	10	328.8	5.406	0.032	87.751	.019
7	{*Ф* (*t*) *p*(*t*)}	15	330.8	7.376	0.012	77.890	
8	{*Ф* (*t*) *p*(c)}	8	330.9	7.505	0.011	94.365	
9	{*Ф* (c) *p*(c)}	2	339.2	15.834	0.000	115.560	

Abbreviations: AICc, Akaike information criterion approx. for small samples; c, constant; NAO, North Atlantic Oscillation Index; np, number of parameters; ONI, Oceanic Niño Index; *p*, recapture probability; *R* ^2^, proportion of explained temporal variation in survival accounted by covariates; *t*, full time dependence (different values for each year); ΔAICc, the difference with the smallest AICc; *Ф*, survival probability.

In the second study period (2002–2012), a sample of 82 breeding Pallid Swifts provided 104 capture events (counting only one recapture per year) (Table S1). The GOF test of the general CJS model *Ф*(*t*)*p*(*t*) suggests that the CJS model's basic assumptions were meet (*χ* ^2^ = 9.143; *df* = 7; *p* = .243), and with a ĉ (*χ* ^2^/*df*) = 1.306, applied to the models. Specific test for transient (U‐Care test 3.SR) was not significant (statistic for transient = 0.484, *p* = .628), but detect a moderate trap dependence (U‐Care TEST2.CT): signed statistic for trap dependence = −2.143, *p* = .032. However, inspecting the test rows, we observed that a trap‐happy effect was only found on the last occasion, and modeling for trap dependence did not affect survival result, while getting unrealistically high capture probabilities. So, we accounted for overdispersion using a ĉ = 1.3.

In the first study period (1984–1992), the model evaluation procedure of the program MARK (results in Table 1) was selected as better models those dependent by the metrics describing the Sahel rainy seasons. Parameters estimated from this model are reported in Table S2.

**FIGURE 2:**
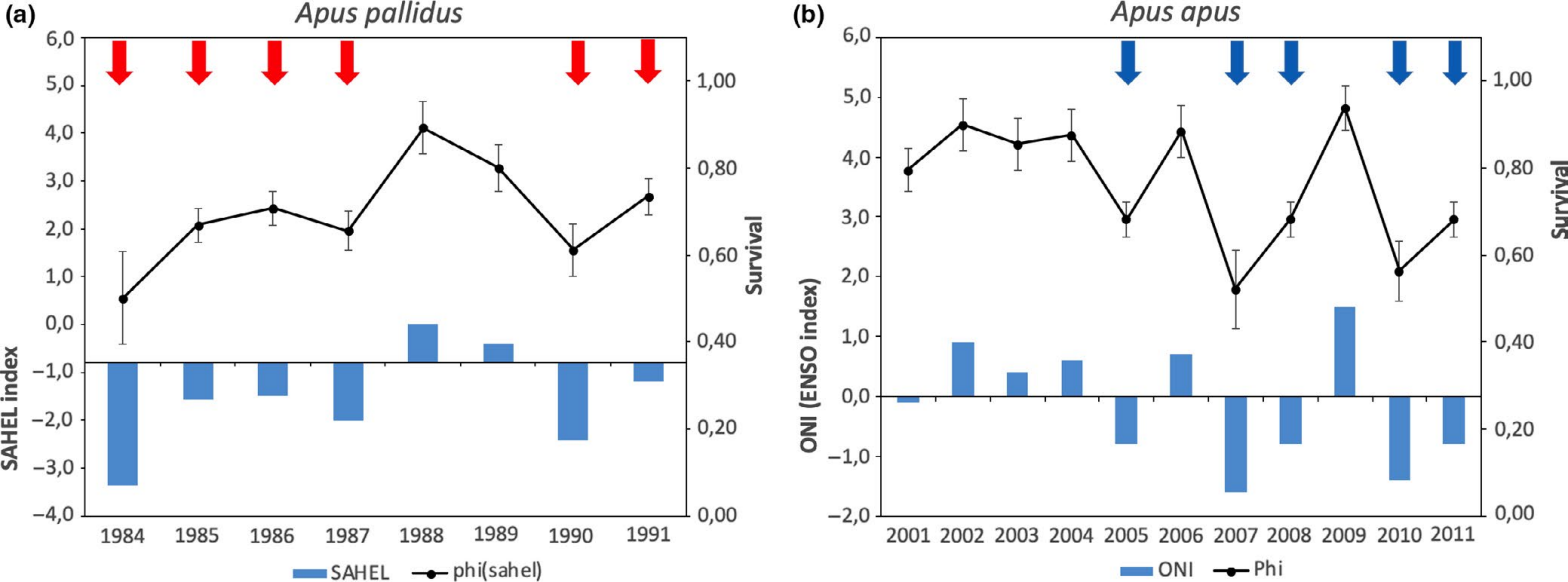
(a) Pallid Swift: variation of annual survival according to the model {*Ф* (Sahel rainfall index) *p*(*t*)} for the years 1984–1991; red arrows point at very dry years. (b) Common Swift: variation of annual survival according to the model {*Ф* (ONI) *p*(*t*)}; blue arrows point “La Niña” (very wet) years. Note the lower survival for the cohorts in 2005, 2007, and 2010

According to the best model, survival probability ranged from 0.62 in the “very dry” season to 0.84 in the “other” seasons. Figure 2a,b shows the survival variability according to the Model 2 (Sahel rainfall index JJASO), with 1984 and 1990 emerging as two particularly worse years. The percentage of annual variation in apparent survival explained by rain variability was 56% (ANODEV *F*_1,6_ = 6.3, *p* = .05).

In the second study period (2002–2012), the model evaluation procedure of the program MARK (results in Table 2) was found as better model the one with constant survival (*Ф* = 0.715; *SE* = 0.075) and variable capture probabilities in the time (Table S3), but the second classified model (ΔQAICc < 2) was still the one considering the “very dry” versus “other” seasons in Sahel, with survival estimates of 0.59 in very dry against 0.87 in”other” years (Table S4). These values were very similar with respect to the ones obtained in 1984–1992. In this case, however, ANODEV indicated that the variable did not add any significant explanation to the interannual variation in adult survival (*F*_1,3_ = 0.416, *p* = .585).

**TABLE 2:** Pallid Swift *Apus pallidus,* period 2002–2012: results of the comparison of the general and related (nested) capture–recapture models (in bold with under delta QAICc < 2)

*N*	Model	np	QAICc	ΔQAICc	Model weights	QDeviance	*R* ^2^
1	**{*Ф* (c) *p(t*)}**	**6**	**102.7**	**0.000**	**0.356**	**30.015**	
2	**{*Ф* (Sahel “very dry” *vs* “other” years) *p*(*t*)}**	**7**	**104.5**	**1.857**	**0.141**	**29.448**	**.172**
3	{*Ф* (ONI) *p*(*t*)}	7	104.8	2.134	0.122	29.726	.088
4	{*Ф* (la Niña) *p*(*t*)}	7	104.8	2.176	0.120	29.767	.075
5	{*Ф* (Sahel rainfall index) *p*(*t*)}	7	105.0	2.328	0.111	29.920	.029
6	{*Ф* (NAO) *p*(*t*)}	7	105.1	2.422	0.106	30.013	.000
7	{*Ф* (*t*) *p*(*t*)}	9	106.9	4.200	0.044	26.728	
8	{*Ф* (c) *p*(c)}	2	116.8	14.140	0.000	53.192	
9	{*Ф* (*t*) *p*(c)}	6	118.8	16.127	0.000	46.142	

Abbreviations: AICc, Akaike information criterion approx. for small samples; c, constant; NAO, North Atlantic Oscillation Index; np, number of parameters; ONI, Oceanic Niño Index; *p*, recapture probability; *R* ^2^, proportion of explained temporal variation in survival accounted by covariates; *t*, full time dependence (different values for each year); ΔAICc, the difference with the smallest AICc; *Ф*, survival probability.

### 3.2 Common Swift

#### 3.2.1 Mark–recapture analysis

A sample of 274 breeding Common Swifts provided 423 capture events (counting only one retrap per individual and year) (Table S1). The GOF test of the general CJS model *Ф*(*t*)*p*(*t*) (*χ* ^2^ = 43.444; *df* = 33; *p* = .106) suggested that the CJS model's basic assumptions were met; ĉ estimated by *χ* ^2^/*df* was 1.316 and this value was used to correct for overdispersion (Cooch & White, 2006). Specific test for transient (U‐Care test 3.SR) and for trap dependence (U‐Care TEST2.CT), were not significant (statistic for transient = 1.081, *p* = .280; signed statistic for trap dependence = 1.051, *p* = .293).

The model evaluation procedure of the program MARK (results in Table 3) was selected as better models (<2 QAICc) the two dependent on ENSO indexes, and time effects on capture probabilities, showing a strong support for the hypothesis that winter climate affects Common Swift survival. The best model, showing the lowest QAICc, was the model that correlated the swift survival with the La Niña years (model 1 in Table 3) resulting in a survival estimate of 0.59 (*SE* 0.06) in “bad” (i.e., La Niña) years versus 0.90 (*SE* 0.06) in all other years. Result of the second best model calculated according to ONI values (Table S5) is highlighted in Figure 2a,b showing 2008 and 2011 as two particularly bad years, with higher mortality. The percentage of annual variation in apparent survival explained by the climate covariable was 72% (ANODEV *F*_1,6_ = 13.0, *p* = .015).

**TABLE 3:** Common Swift *Apus apus,* period 2001–2012: results of the comparison of the general and related (nested) capture–recapture models (in bold results with ΔQAICc < 2)

*N*	Model	np	QAICc	ΔQAICc	QAICc Weights	QDeviance	*R* ^2^
**1**	**{*Ф* (la Niña years) *p*(*t*)}**	**13**	**591.1**	**0.000**	**0.580**	**126.629**	**.723**
**2**	**{*Ф* (ONI) *p*(*t*)}**	**13**	**592.9**	**1.766**	**0.240**	**128.396**	**.554**
3	{*Ф* (Sahel “very dry” versus “other” years) *p*(*t*)}	13	595.0	3.831	0.085	130.460	.357
4	{*Ф* (c) *p*(*t*)}	12	596.6	5.408	0.039	134.198	
5	{*Ф* (Sahel rainfall index) *p*(*t*)}	13	597.0	5.817	0.032	132.446	.167
6	{*Ф* (NAO) *p*(*t*)}	13	598.6	7.498	0.014	134.127	.007
7	{*Ф* (*t*) *p*(*t*)}	18	599.2	8.095	0.010	123.723	
8	{*Ф* (*t*) *p*(c)}	8	606.5	15.382	0.000	152.684	
9	{*Ф* (c) *p*(c)}	2	622.9	31.725	0.000	181.422	

Abbreviations: AICc, Akaike information criterion approx. for small samples; c, constant; NAO, North Atlantic Oscillation Index; np, number of parameters; ONI, Oceanic Niño Index; *p*, recapture probability; *R* ^2^, proportion of explained temporal variation in survival accounted by covariates; *t*, full time dependence (different values for each year); ΔAICc, the difference with the smallest AICc; *Ф*, survival probability.

#### 3.2.2 Nonbreeding movements tracked by geolocation

**FIGURE 3:**
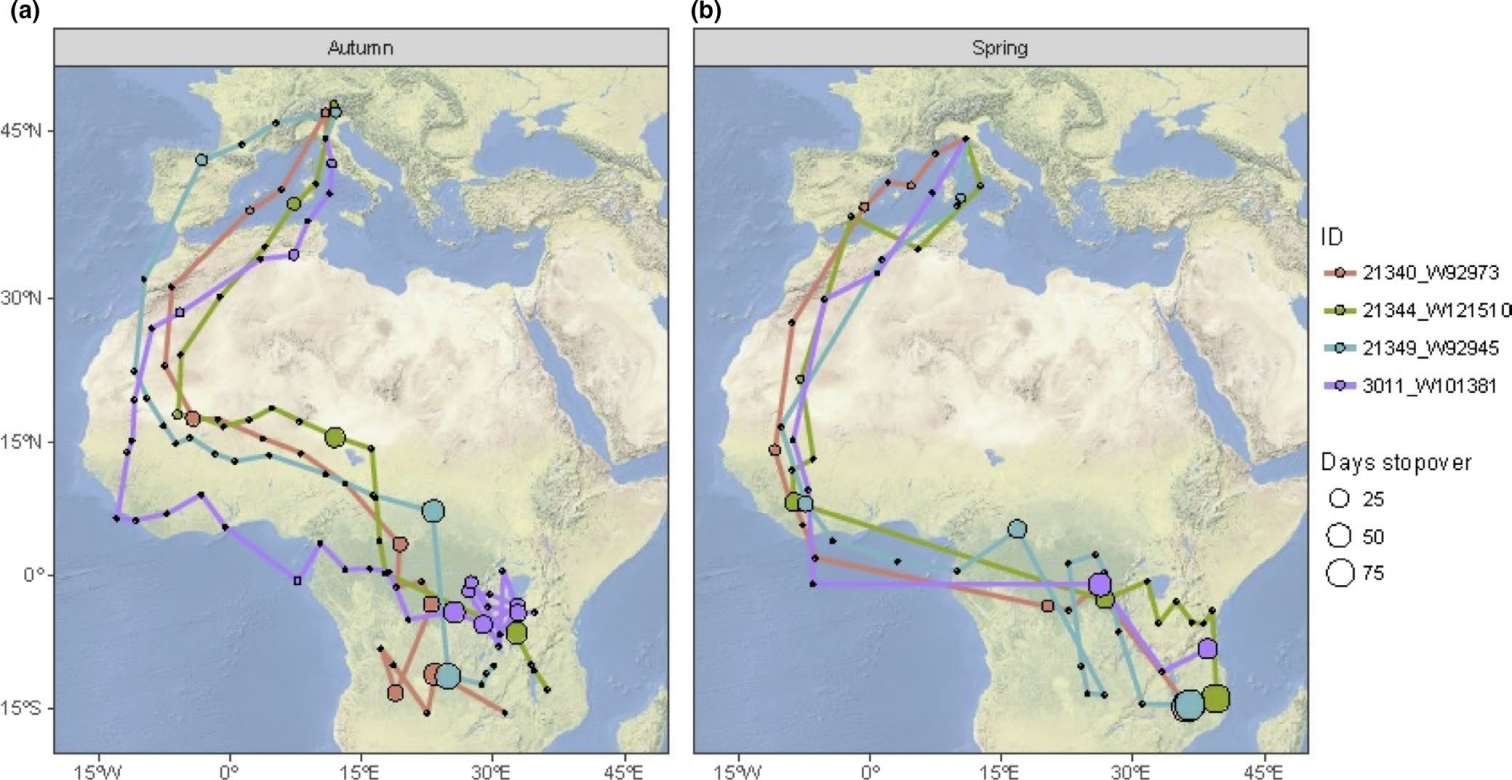
Nonbreeding movements (black dots: 2‐day averages) and location of extended stopover periods (filled circles) in autumn (a) and spring (b) for Common Swifts tracked by geolocation from northern Italy. Tracking data are color‐coded for individual birds

We were able to track the nonbreeding movements of four adult breeding Common Swifts from our study colony in Modena (3 individuals 2010–2011; 1 individual 2012–2013; Figure 3). The tracking data reveal that the swifts departed from the breeding site 7–14 July (median date: 14 July) and returned the next year to the breeding sites 12 April to 5 June (median date: 27 April). The swifts arrived at the first wintering sites in the Sahel zone south of the Sahara by 4–8 August (median date: 5 August). The individuals moved between several locations where they were stationary for extended periods (>3 days) of time during the nonbreeding period, and prolonged times were spent in areas in the Sahel zone, central Africa during the initial part of the winter, and thereafter in central and southeastern Africa in Midwinter (Figure 3). In spring, several of the swifts were stationary for a short period of time in West Africa before they crossed the Sahara north to the breeding area (Figure 3).

## 4 DISCUSSION

### 4.1 Survival probabilities for adult swifts

Swift are long‐lived birds, and indeed, the survival of Apodiformes is very high when compared to passerine aerial feeders such as swallows (Ferro & Boano, 1998; Masoero, Tamietti, Boano, & Caprio, 2016). In this study, we found a mean annual survival similar to those reported in the previous studies available, both for the Common (0.78) and the Pallid Swift (ranging from 0.71 to 0.76). A correct comparison of the survival values reported in different studies should, however, consider the methodology employed for data collection and analysis. In some studies, the method involves the use of ringing recoveries, that is, the observations of all birds that were found dead or alive in a vast area (UK scheme: Baillie & Green, 1987; Dobson, 1990). In other studies, the method involved the use of recaptures, that is, observations based on capture–mark–recapture of live individuals in the same locality. The values of survival obtained by only recapture data should be considered as apparent (or “local”) survival. The method can slightly underestimate the global survival, because some individuals that were alive can instead be considered as dead if they move (emigrate) to a different and not examined site. In the particular case of the Pallid and Common Swift, the difference between the recovery and recapture methods is likely very small. The adult swifts show a high philopatry and fidelity to their breeding colony, and even to the single nest (Boano et al., 1993; Lack, 1956; Weitnauer, 1980). Hence, the difference in estimates related to individuals moving to other sites should be minimal.

The higher mean value reported by Baillie and Green (1987) for Common Swift is probably due to the fact that this study is one of the few taking into account recoveries over the entire species range and not only from live recaptures at a single colony. In effect, a similar recovery analysis by Dobson (1990) obtained a lower survival value (0.76); the same values obtained by Lebreton et al. (1992) and Thomson et al. (1996) with live recaptures and the survival estimates for Pallid Swift are strictly comparable or eventually a bit lower (but not statistically significant) (Table 4).

**TABLE 4:** Apparent survival estimates for Common and Pallid Swifts (obtained with recoveries or retrievals) in different countries

Country	Years	*Φ* (±*SE*)	*N*	Model	Authors
Common Swift—All recoveries					
(1) Great Britain	1954–1966	0.79	162	H	Perrins (1971)
(2) Great Britain	1956–1975	0.81 ± 0.3^a^	2,587	*Φ*t	Baillie and Green (1987)
(3) Great Britain	1966–1978	0.76 ± 0.3	111	H	Dobson (1990)
Common Swift—Local live recapture					
(1) Great Britain	1958–1968	0.84	60	H	Perrins (1971)
(2) Great Britain	1954–1993	0.76 ± 0.02^a^	—	*Φ*t	Thomson et al. (1996)
(3) France (good)	1982–1989	0.76 ± 0.04	88	*Φ*e	Lebreton et al. (1992)
(poor)		0.62 ± 0.08	39	*Φ*e	
(4) France (good	1982–1993	0.78 ± 0.03	—	*Φ*e	Viallefont et al. (1998)
(poor 1)		0.65 ± 0.05	—	*Φ*e	Viallefont et al. (1998)
(poor 2)		0.52 ± 0.06	—	*Φ*e	Viallefont et al. (1998)
(5) Italy (good)	2001–2012	0.90 ± 0.06	274	*Φ*e	This study
(poor)		0.59 ± 0.06		*Φ*e	
		0.78^a^		*Φ*t	
Pallid Swift—Local live recaptures					
(6) Gibraltar (UK)		0.74	—	H	Finlayson (1979)
(7) Portugal (good)	1990–1996	0.86	135	*Φ*e	Costa and Elias (1998)
(poor)		0.75		*Φ*e	Costa and Elias (1998)
(8) Italy	1987–1992	0.76 ± 0.06	78	*Φ*c	Boano et al. (1993)
(9) Italy (good)	1984–1992	0.84 ± 0.06	106	*Φ*e	This study
(poor)		0.62 ± 0.05		*Φ*e	Costa and Elias (1998)
		0.72^a^		*Φ*t	
(10) Italy	2002–2012	0.71 ± 0.7	82	*Φ*c	This study

Note

*N*, number of different individual ringed. Survival estimates by CJS models *Φt*, time‐dependent model; *Φ*c, CJS constant model; *Φ*e, model depending on effect (“poor” or “good” climate and/or colony conditions according to the Authors) and by Haldane (1955) method (H) assuming constant survival and capture probabilities.

^a^ Average of different annual survival estimates based on *Φt* model.

The uniformity of survival values found both within different latitude populations of the same species of swift, and between our two species examined here, is someway unexpected. The observation of latitudinal gradients in bird life‐history traits has strongly affected the study of avian life‐history patterns and evolution (Lack, 1947). The well‐documented latitudinal trend in clutch size or number of clutches per year (Jetz, Sekercioglu, & Böhning‐Gaese, 2008) was also found in our study species, where the Common Swift has a northern distribution range and lay a single clutch, while the Pallid Swift with a southern range most often lay two clutches per season. In a perspective of fecundity‐survival trade‐off (Sæther, 1988), the latitudinal gradient in number of clutches should be matched by an inverse trend in survival probability. Indeed, some studies reported observations that temperate birds have lower survival probabilities than their tropical counterparts (Martin, 1996; Murray, 1985; Skutch, 1985). However, the idea of higher adult survival at lower latitudes is still debated and questions have arisen concerning a true statistical support of such a gradient (Pizarro Muñoz, Kéry, Martins, & Ferraz, 2018).

### 4.2 Wintering areas and movement of common and pallid swifts

The geolocation tracking by Commons Swift in this study showed that the birds performed their migration earlier in autumn as compared to more northern populations (Åkesson et al., 2012, 2016; Hedenström et al., 2016) and that their final winter destination were located further to the southeast in Africa (Figure 3). The locations of the nonbreeding areas were further to the east as compared to commons swifts tracked from breeding sites in Germany (Wellbrock, Bauch, Rozman, & Witte, 2017). The Common Swifts, moreover, explored several geographic areas during stationary periods in sub‐Saharan Africa on their way to the final winter destinations, with the first stops lasting 1–3 weeks occurring in the Sahel zone. However, during midwinter in November to February the Common Swifts from our study colony also expressed somewhat more nomadic exploratory movements, with stationary phases mixed with movement phases, as compared to Common Swifts tracked in Sweden (Åkesson et al., 2012). The Pallid Swifts depart on autumn migration much later than the Commons Swifts and will therefore arrive to the wintering areas in western Africa much later. The Pallid Swifts will, furthermore, perform much shorter intertropical movements during winter than the Common Swifts. These movements by Pallid Swifts from our study colony are presented in detail in Norevik et al. (2019), showing stops in the Sahel zone, but also shifts in area use following the local rain patterns toward south in winter. Both species spend most of their nonbreeding period on the wing lasting up to 10 months for the Common Swift (Hedenström et al., 2016) and 5 months for the Pallid Swift (Hedenström et al., 2019), respectively.

The only previous work that suggests where the north Italian Common Swifts spend their winter pose on an indirect estimation, correlating the number of swift pairs censused with the point count method in Lombardy with African climate indexes. The authors of this study inferred that Common Swifts go to Ghana, in West Africa (Ambrosini, Orioli, Massimino, & Bani, 2011). This, however, seems to contrast what is known on the wintering of Common Swift (Del Hoyo, Elliot, & Sargatal, 1999), and differs considerably from our data presented here collected with geolocators. We suggest that the indirect method employed by Ambrosini et al. (2011) cannot be safely utilized to estimate wintering areas of swifts, but we should in the future preferably rely on tracking data such as those based on geolocation presented here to infer nonbreeding areas for swifts and other migratory species.

### 4.3 Relationships between survival and winter climate in Africa

In our study, we found a strong difference of climatic factors influencing survival when comparing Common and Pallid Swifts breeding in the same area. While Pallid Swift survival was related to the Sahel wet/dry index, and the Common Swift survival was related to the ENSO cycle measured with the ONI. The data collected with the geolocators support the finding that both species spent their nonbreeding period exactly in the areas pertaining to the significant indices, suggesting the wintering period is a critical period for survival in these swifts.

The Pallid Swift spend the winter flying continuously (Hedenström et al., 2019) over a range of habitats in West Africa. It seems like the Sahel rainfall is influencing the survival even if the birds can adjust their winter area following the rain by progressively moving south during the nonbreeding season (Norevik et al., 2019). The data collected for Pallid Swift during the second period of our study, from 2002 to 2012, are less straightforward, pointing at best model to the constant survival. However, even in this case the second best model point on a Sahel rain effect simply scaled among “very dry” and “other” condition. Probably, this result could be related to the recent rainfall recovery with lack of extreme dry events in this 11‐year period (Munemoto & Tachibana, 2012; Sanogo et al., 2015). Similar effects of weather were previously found in other species wintering in Western Africa, for example, the Common Nightingale (*Luscinia megarhynchos*) wintering in Guinean coast influenced on the way of return to Europe by a negative effect of the very dry seasons (Boano, Bonardi, & Silvano, 2004).

The Common Swifts from our study colony seems to winter mainly in Mozambique and nearby countries in southeast Africa, being strongly affected by the ENSO cycle. The ENSO is a phenomenon that originates in the Pacific Ocean with years in which the current is warmer than usual (El Niño) and years when it is colder (La Niña). Various studies of climatologists have found worldwide effects, including some in Africa. For example, in the years of La Niña, in southeast Africa there are often exceptional rains and exaggerated flooding, while a little further north in East Africa there can be droughts instead (Figure 3). According to our study, mortality of our Common Swifts is higher during La Niña years. La Niña involve large shifts of rainfall patterns to the southwest into Australia, Indonesia, and southern Asia. This leaves less rain for eastern African countries including Uganda, Kenya, Ethiopia, and Somalia (Nicholson & Selato, 2000; Schrage et al., 2004), and more south in Africa the precipitation can at the same time be very strong, long‐lasting, and widespread causing very severe and extensive flooding (e.g., in 2008; Lukamba, 2010). The areas affected by these rain anomalies correspond to the areas where we know that our marked swifts are spending their nonbreeding period in winter (see maps in Figure 1).

Summing up, the results from our study suggest that the variable climatic conditions, as found in the wintering area, show evident effect on swift's survival only when the adverse effects exceed a certain limit. In this scenario, the swifts’ survival is mostly affected when particularly worse conditions, that is, extreme drought or heavy rainfall, occur in the two major regions explored in Africa by the two species, respectively.

## 5 CONCLUSIONS

We have found that two species of swifts that differ in biological traits and wintering area used show similar yearly survival, but are influenced by different climatic drivers, resulting in annual difference in survival. We could confirm that the important climatic variables can be predicted, since precise winter locations are known for our study populations. It is interesting to note that adult average annual survival rate is similar between the two species despite differences in migratory strategy, wintering areas, and breeding biology, with a double brood in Pallid and single brood in Common Swift. The difference in breeding investment between the two species, involving a longer period spent in the breeding areas for Pallid Swifts as compared to the Common Swifts, is perhaps balanced by a shorter migration in Pallid Swifts. Future studies, however, need to investigate why two highly mobile aerial insectivores spending the complete nonbreeding period on the wing are not able to escape from extreme weather conditions by changing wintering area, even if they may be capable of some adjustment as showed by Åkesson et al. (2012) and Norevik et al. (2019).

## CONFLICT OF INTEREST

The authors declare no conflicts of interest.

## AUTHOR CONTRIBUTION

**Giovanni Boano:** Conceptualization (supporting); Investigation (equal); Methodology (equal); Writing‐original draft (supporting). **Irene Pellegrino:** Investigation (equal); Writing‐original draft (equal); Writing‐review & editing (supporting). **Mauro Ferri:** Investigation (equal). **Marco Cucco:** Data curation (supporting); Investigation (equal); Writing‐original draft (equal). **Fausto Minelli:** Investigation (equal). **Susanne Åkesson:** Conceptualization (supporting); Data curation (supporting); Formal analysis (supporting); Investigation (supporting); Methodology (supporting); Project administration (lead); Resources (supporting); Writing‐original draft (supporting); Writing‐review & editing (supporting).

## PERMITS

Permission to ring and work with swifts in Italy was given by ISPRA (INFS) and Città Metropolitana di Torino to Giovanni Boano, and by Provincia di Modena (Prot. n. 18185/19.02.2015) to Fausto Minelli.

## Supporting information

Tables S1‐S6

## Data Availability

The GLS data about that support the findings of this study are available in the CAnMove (Centre for Animal Movement Research) database www.canmove.lu.se and in the Dryad Digital Repository: https://doi.org/10.5061/dryad.f5dv1t0 (Hedenström et al., 2019).
