# Supplementary material for: Climate anomalies affect annual survival rates of swifts wintering in sub‐Saharan Africa
Source: Ecol Evol. 2020 Jul 6;10(14):7916–28. doi: 10.1002/ece3.6525 (PMC7391547; doi:10.1002/ece3.6525)
Supplement: Supplementary file 1 — Tables S1‐S6 [file ECE3-10-7916-s001.docx]

**Supplementary material**

**Table S1** - Number of individual Pallid and Common Swifts breeding adults released (newly ringed or controlled) each year.

________________________________________________________________________________

*Apus pallidus*

YEAR 1984 1985 1986 1987 1988 1989 1990 1991 1992

Realesed 23 11 8 6 23 29 27 35 32

YEAR 2002 2003 2004 2005 2006 2007 2008 2009 2010 2011 2012

Realesed 6 3 2 6 8 19 0 6 9 18 27

*Apus apus*

YEAR 2001 2002 2003 2004 2005 2006 2007 2008 2009 2010 2011 2012

Realesed 25 9 28 34 21 36 41 3 38 60 49 79

________________________________________________________________________________

**Table S2** - Pallid Swift *Apus pallidus,* period 1984-1992*.* Annual parameter values after the model *Ф* {(Sahel “very dry” *vs* “other” years) p(t)}. *Ф* = survival probability; *p* = recapture probability; SE = Standars error; LCL = lower confidence limit; UCL = upper confidence limit.

| Label | Estimate | SE | LCL | UCL |
| --- | --- | --- | --- | --- |
| *Ф* (very dry years) | 0.621 | 0.055 | 0.509 | 0.721 |
| *Ф* (other years) | 0.837 | 0.061 | 0.681 | 0.925 |
| *p* (1985) | 0.345 | 0.135 | 0.140 | 0.630 |
| *p* (1986) | 0.082 | 0.080 | 0.011 | 0.419 |
| *p* (1987) | 0.253 | 0.133 | 0.078 | 0.574 |
| *p* (1988) | 0.662 | 0.155 | 0.336 | 0.884 |
| *p (*1989) | 0.653 | 0.113 | 0.415 | 0.833 |
| *p* (1990) | 0.703 | 0.095 | 0.492 | 0.853 |
| *p* (1991) | 0.881 | 0.103 | 0.518 | 0.981 |
| *p* (*1*992) | 0.729 | 0.155 | 0.366 | 0.926 |

**Table S3** - Pallid Swift *Apus pallidus,* period 2002-2012, annual parameter values after the model *Ф*(c) *p*(t). *Ф* = survival probability; *p* = recapture probability; SE = Standard error; LCL = lower confidence limit; UCL = upper confidence limit.

| Index | Label | Estimate | SE | LCL | UCL |
| --- | --- | --- | --- | --- | --- |
| 1 | *Ф* | 0.715 | 0.075 | 0.550 | 0.838 |
| 2 | *p*2003 | 0.000 | 0.000 | 0.000 | 0.000 |
| 3 | *p*2004 | 0.000 | 0.000 | 0.000 | 0.001 |
| 4 | *p*2005 | 0.000 | 0.000 | 0.000 | 0.001 |
| 5 | *p*2006 | 0.000 | 0.000 | 0.000 | 0.000 |
| 6 | *p*2007 | 0.656 | 0.235 | 0.198 | 0.936 |
| 7 | *p*2008 | 0.000 | 0.000 | 0.000 | 0.015 |
| 8 | *p*2009 | 0.086 | 0.088 | 0.010 | 0.462 |
| 9 | *p*2010 | 0.252 | 0.150 | 0.066 | 0.616 |
| 10 | *p*2011 | 0.304 | 0.156 | 0.093 | 0.650 |
| 11 | *p*2012 | 0.360 | 0.146 | 0.140 | 0.661 |

**Table S4 -** Pallid Swift *Apus pallidus,* period 2002-2012*.* Annual parameter values after the model {*Ф*(“very dry” *vs* “other” years) *p*(t)}. *Ф* = survival probability; *p* = recapture probability; SE = Standard error; LCL = lower confidence limit; UCL = upper confidence limit.

| Label | Estimate | SE | LCL | UCL |
| --- | --- | --- | --- | --- |
| *Ф* (very dry years) | 0.589 | 0.157 | 0.287 | 0.836 |
| *Ф* (other years) | 0.868 | 0.221 | 0.129 | 0.997 |
| *p* (2003) | 0.000 | 0.000 | 0.000 | 0.194 |
| *p* (2004) | 0.000 | 0.000 | 0.000 | 0.034 |
| *p* (2005) | 0.000 | 0.000 | 0.000 | 0.104 |
| *p* (2006) | 0.000 | 0.000 | 0.000 | 0.569 |
| *p* (2007) | 0.918 | 0.480 | 0.000 | 1.000 |
| *p* (2008) | 0.000 | 0.000 | 0.000 | 0.001 |
| *p*(2009) | 0.100 | 0.104 | 0.011 | 0.515 |
| *p* (2010) | 0.233 | 0.142 | 0.060 | 0.591 |
| *p* (2011) | 0.243 | 0.150 | 0.061 | 0.614 |
| *p* (2012) | 0.391 | 0.172 | 0.134 | 0.726 |

**Table S5.** Common Swift *Apus apus,* annual parameter values estimate according the model {*Ф*(ONI) *p*(t)}. *Ф* = survival probability; *p* = recapture probability; SE = Standard error; LCL = lower confidence limit; UCL = upper confidence limit.

| Index | Label | Estimate | SE | LCL | UCL |
| --- | --- | --- | --- | --- | --- |
| 1 | *Ф*2001 | 0.795 | 0.05 | 0.68 | 0.87 |
| 2 | *Ф*2002 | 0.900 | 0.06 | 0.72 | 0.97 |
| 3 | *Ф* 2003 | 0.855 | 0.06 | 0.70 | 0.94 |
| 4 | *Ф* 2004 | 0.875 | 0.06 | 0.71 | 0.95 |
| 5 | *Ф* 2005 | 0.682 | 0.04 | 0.61 | 0.75 |
| 6 | *Ф* 2006 | 0.884 | 0.06 | 0.71 | 0.96 |
| 7 | *Ф* 2007 | 0.521 | 0.09 | 0.35 | 0.69 |
| 8 | *Ф* 2008 | 0.682 | 0.04 | 0.61 | 0.75 |
| 9 | *Ф* 2009 | 0.938 | 0.05 | 0.73 | 0.99 |
| 10 | *Ф* 2010 | 0.563 | 0.07 | 0.42 | 0.7 |
| 11 | *Ф* 2011 | 0.682 | 0.04 | 0.61 | 0.75 |
| 12 | *p*2002 | 0.047 | 0.05 | 0.00 | 0.34 |
| 13 | *p*2003 | 0.378 | 0.12 | 0.19 | 0.61 |
| 14 | *p*2004 | 0.320 | 0.1 | 0.16 | 0.53 |
| 15 | *p*2005 | 0.115 | 0.05 | 0.04 | 0.27 |
| 16 | *p*2006 | 0.247 | 0.08 | 0.12 | 0.44 |
| 17 | *p*2007 | 0.358 | 0.09 | 0.21 | 0.54 |
| 18 | *p*2008 | 0.049 | 0.04 | 0.01 | 0.21 |
| 19 | *p*2009 | 0.362 | 0.12 | 0.17 | 0.62 |
| 20 | *p*2010 | 0.288 | 0.08 | 0.16 | 0.46 |
| 21 | *p*2011 | 0.281 | 0.08 | 0.16 | 0.45 |
| 22 | *p*2012 | 0.717 | 0.12 | 0.43 | 0.89 |

**Table S6:** Analyses of Deviation (ANODEV)

**Pallid Swift, Carmagnola (Turin), 1984-1992**

Global Model: {*Ф*(t) *p*(t)}

Constant Model: {*Ф*(c) *p*(t)}

Covariate Model: {*Ф*(Sahel “very dry” *vs* “other” years) *p*(t)}

Source df Dev Mean Dev F P

=============================================================

Uncorrected Total 15 87.939

Grand Mean 9 77.890

Corrected Total 6 10.049

Total Covariate 1 5.595 5.595 6.2802 0.0541

Error 5 4.454 0.891

=============================================================

**Pallid Swift, Carmagnola (Turin), 2002-2012**

Global Model: {*Ф*(t) *p*(t)}

Constant Model: {*Ф*(c) *p*(t)}

Covariate Model: {*Ф*(Sahel “very dry” *vs* “other” years) *p*(t)}

Source df QDev Mean QDev F P

=============================================================

Uncorrected Total 9 30.015

Grand Mean 6 26.728

Corrected Total 3 3.287

Total Covariate 1 0.566 0.566 0.4164 0.5849

Error 2 2.720 1.360

=============================================================

**Common Swift, Castellaro (Modena), 2001-2012**

Global Model: {*Ф*(t) *p*(t)}

Constant Model: {*Ф*(c) *p*(t)}

Covariate Model: {*Ф*(la Nina years) *p*(t)}

Source df QDev Mean QDev F P

=============================================================

Uncorrected Total 18 134.198

Grand Mean 12 123.723

Corrected Total 6 10.475

Total Covariate 1 7.569 7.569 13.0210 0.0154

Error 5 2.906 0.581

=============================================================
